# Intraoperative and Postoperative Complications in Gynaecological Surgery: A Retrospective Analysis

**DOI:** 10.7759/cureus.14885

**Published:** 2021-05-07

**Authors:** Anupama Bahadur, Rajlaxmi Mundhra, Jyotshna Kashibhatla, Latika Chawla, Megha Ajmani, Shloka Sharma, Rabia Zaman, Modalavalasa Swetha Sri

**Affiliations:** 1 Obstetrics and Gynaecology, All India Institute of Medical Sciences, Rishikesh, Rishikesh, IND

**Keywords:** gynaecology, surgical outcomes, perioperative complications, surgical site infections

## Abstract

Background and objective

Surgical complications can arise either intraoperatively or postoperatively. The factors that lead to complications in gynaecological surgeries could be both patient-related or surgeon-related. In this study, we aimed to identify the frequency of intraoperative and postoperative complications in gynaecological surgeries conducted at our institution and to evaluate various risk factors that may predispose patients to these complications.

Materials and methods

This was a retrospective analysis of women undergoing gynaecological surgeries in the Department of Obstetrics and Gynaecology at a tertiary centre in Uttarakhand, India from February 2016 to December 2019. Demographic characteristics, comorbidities, and perioperative complications of these women were recorded.

Results

A total of 389 women undergoing gynaecological surgeries were included in the study cohort. Of note, 94 of these had perioperative complications, accounting for 24.16% of the total cases. The most common route of surgery associated with complications was open abdominal surgery (34.66%). The operating time in most surgeries ranged from two to three hours (48.93%), and the average duration of hospital stay after surgery was 10.79 + 7.91 days. Intraoperative and postoperative complication rates were 5.91% and 19.28% respectively. Of these, surgical site infections (SSIs) (10.28%) and fever (5.39%) were the most common complications observed. Independent parameters like age, parity, route of surgery, operative time, preoperative duration of hospital stay, and preoperative blood transfusion were significantly associated with perioperative complications.

Conclusions

Surgical complications were more frequently seen in abdominal cases compared to other routes. Knowledge of centre-specific surgical outcome data can help in providing patients with better preoperative counselling.

## Introduction

All surgical procedures have certain risks associated with them. These complications can arise both intraoperatively or postoperatively. Ureters, bowels, vessels, and nerves are in close proximity to the uterus, making them prone to injuries. Intraoperative complications can be repaired immediately if they are promptly identified during the surgery. Hence, in-depth knowledge of pelvic anatomy is an essential prerequisite for the operating surgeon for early recognition and repair, thereby minimising sequelae. It is estimated that gynaecological surgeries account for 75% of iatrogenic injuries to the urinary tract [1,2]. Nearly 0.2-1% of gynaecological surgeries can be complicated by urinary tract injuries [1,3]. The most common surgeries performed in gynaecology are hysterectomy, salpingectomy, and cystectomy [4]. These surgeries can be performed either by the abdominal or vaginal route, by open technique, or by minimally invasive techniques such as laparoscopy and robotic methods. Also, staging laparotomy and radical hysterectomy are performed for carcinoma of the ovary, endometrium, and cervix. Each of these routes of surgery carries its own advantages and disadvantages.

Postoperative complications, on the other hand, can arise at any time following the surgery. These can be diverse and include fever, upper respiratory infections, surgical site infections (SSIs), urinary tract infections, urinary retention, and abdominal distension. SSIs account for 0.5-15% of the total postoperative complications and are the most commonly encountered ones [5]. According to World Health Organization (WHO), it complicates 11.8 out of 100 surgical procedures in low- and middle-income countries [6]. These have emerged as one of the costliest causes of healthcare-associated infections [7]. The factors that lead to these unwanted complications could be either patient-related or surgeon-related. In the intraoperative setting, ascites, inflamed bowel, distorted anatomy, previous surgery, endometriosis, carcinomas, and pelvic inflammatory disease can increase the risk of injury. The patient’s age, comorbidities, weight, level of compliance, hygiene, nutrition, and functional performance may prove to be directly or indirectly causative. Similarly, the sterility of the operation theatre and operating staff, aseptic conditions, and surgical errors can also result in complications [5].

These complications can sometimes pose legal problems to the surgeon. Timely diagnosis and prompt treatment are key to dealing with these problems. Moreover, keen awareness and insight into complications are a must so that proper patient counselling can be carried out. A retrospective evaluation of the details of these procedures that focus on the mistakes that occurred can give an insight into the subject so that similar errors can be avoided in the future. In this article, we aimed to identify the frequency of intraoperative and postoperative complications in gynaecological surgeries conducted at our institution and analyse the various risk factors that may predispose patients to these complications.

## Materials and methods

This was a single-institution retrospective study carried out at a tertiary centre in Uttarakhand, India from February 2016 to December 2019. The clinical records of the patients undergoing gynaecological surgeries by any route in the Department of Gynaecology were included in the study. Patients who were pregnant, those with incomplete data, and those who underwent emergency gynaecological surgeries were excluded from the research. Outcome variables included any complication reported intraoperatively or within 30 days post-surgery.

All surgeries were performed by an experienced surgeon. Patient details including the duration of surgery, route of surgery, duration of hospital stay, and intraoperative and postoperative complications were recorded in a proforma. An Excel spreadsheet was prepared with all these details. We also evaluated the association between the presence of complications and parameters like age, parity, the approach of surgery, duration of surgery, preoperative duration of hospital stay (days), as well as preoperative blood transfusion. Descriptive statistics, chi-square test, Wilcoxon Mann-Whitney U test, and Fisher's exact test were performed as appropriate.

## Results

A total of 389 patients were operated on by abdominal, vaginal, and laparoscopic routes during the study period, from February 2016 to December 2019. There were 94 cases with intraoperative or postoperative complications, accounting for 24.16% of the cohort. The mean age of patients with and without complications was 43.06 ± 11.97 and 40.14 ± 12.96 years respectively. The majority of women in both groups were postmenopausal (almost 78%). Approximately 78.72% of patients with complications had no associated comorbidities (Table 1).

**Table 1:** Baseline characteristics of the patients SD: standard deviation

Parameters	Complications	
	Present (n=94)	Absent (n=295)
Age (years), mean ± SD	43.06 ± 11.97	40.14 ± 12.96
Premenopausal; postmenopausal, n (%)	74 (78.72%); 20 (21.27%)	231 (78.30%); 64 (21.69%)
Comorbidities absent, n (%)	74 (78.72%)	259 (87.79%)
Hypertension, n (%)	6 (6.38%)	14 (4.74%)
Hypothyroidism, n (%)	7 (7.44%)	8 (2.71%)
Diabetes mellitus, n (%)	2 (2.12%)	9 (3.05%)
Multiple comorbidities, n (%)	5 (5.31%)	5 (1.69%)

As seen in Table 2, the most common route of surgery associated with complications was open abdominal surgery (34.66%), followed by vaginal surgeries (14.49%), and laparoscopic surgeries (6.38%). The most common indication for surgery was abnormal uterine bleeding (40.42%). This was followed by adnexal masses (21.2%), uterocervical or vault prolapse (18.08%), carcinoma endometrium (9.57%), carcinoma ovary (8.51%), and ruptured ectopic pregnancy (2.12%). One case of anterior vaginal wall mass was also seen, which was densely adhered to the posterior wall of the bladder and neck of the urethra, leading the bladder and urethra to get injured during its excision. The surgery associated with the most number of complications was total abdominal hysterectomy + bilateral salpingo-oophorectomy (45.74%), followed by staging laparotomy (15.95%), and exploratory laparotomy (11.70%). The longest median duration of surgery (for those with complications) was associated with the abdominal route (three hours), followed by the vaginal route (two hours), and the laparoscopic route (1.7 hours). The mean duration of hospital stay after surgery was 11.50 days in abdominal surgeries, 7.50 days in vaginal surgeries, and 7.30 days in laparoscopic surgeries.

**Table 2:** Route of surgery in patients with complications (n=94) Ca: carcinoma; BSO: bilateral salpingo-oophorectomy

Variables	Values	Percentage
Total surgeries, surgical complications/number of surgeries	94/389	24.16%
Surgical approach, surgical complications/number of surgeries		
Abdominal surgeries	78/225	34.66%
Vaginal surgeries	10/69	14.49%
Laparoscopic surgeries	6/95	6.31%
Diagnosis of cases with complications, n		
Abnormal uterine bleeding	38	40.42%
Adnexal masses	20	21.2%
Pelvic organ prolapse	17	18.08%
Ca endometrium	9	9.57%
Ca ovary	8	8.51%
Ca cervix	3	3.19%
Ruptured ectopic pregnancy	2	2.12%
Molar pregnancy	1	1.06%
Anterior vaginal wall mass	1	1.06%
Surgical procedures, n		
Abdominal surgeries	78	82.97%
Total abdominal hysterectomy + BSO	43	45.74%
Staging laparotomy	15	15.95%
Exploratory laparotomy	11	11.70%
Myomectomy	5	5.31%
Radical hysterectomy	2	2.12%
Sacrocolpopexy	2	2.21%
Vaginal surgeries	10	10.63%
Vaginal hysterectomy	8	8.51%
Suction and evacuation	1	1.06%
Vaginal mass excision	1	1.06%
Laparoscopic surgeries	6	6.38%
Diagnostic hysterolaparoscopy	1	1.06%
Total laparoscopic hysterectomy	1	1.06%
Laparoscopic cystectomy	4	4.25%
Median duration of surgery, hours		
Abdominal surgeries	3	
Vaginal surgeries	2	
Laparoscopic surgeries	1.7	
Mean duration of hospital stay, days		
Abdominal surgeries	11.50	
Vaginal surgeries	7.50	
Laparoscopic surgeries	7.30	

As shown in Table 3, 5.91% of surgeries met with complications intraoperatively whereas 19.28% of surgeries presented complications in the postoperative period. Intraoperative haemorrhage (characterised by a change in vitals and/or need for blood and blood products transfusion) was one of the most common complications encountered intraoperatively, accounting for 14 cases (3.59%). In all these cases, transfusion of blood and various blood products as per need were imperative. Urological injuries constituted 1.02% of cases, which included urethral injury (0.25%) and bladder injury (0.77%). There was one case of bowel serosal injury (0.25%), which was identified and repaired intraoperatively. The inferior epigastric artery was injured in one case while creating a port for laparoscopic surgery. It was identified immediately with vessel ligation. Anaphylactic shock was seen in a case of laparoscopic cystectomy just after induction with general anaesthesia. There were two patients (0.51%) who had arrhythmia during the surgery. Both these patients had normal ECG preoperatively and had no history of cardiac disease. 

SSI was the most common major postoperative complication and was seen in 40 patients, accounting for 10.28% of cases. These patients presented on postoperative days two or three with discharge from the stitch line (38 patients) or discharge per vaginum (four patients). Two patients presented with both complaints. Discharge from the stitch line and high vaginal swab were sent for culture and sensitivity in these patients as per our protocol. Daily dressing and vaginal douching were performed, and antibiotics were started as per the sensitivity report. Resuturing was done after the wound was healthy in 18 patients out of 38 (47.3%). Two patients out of 38 (5.26%) underwent re-laparotomy due to a burst abdomen. Fever was the next most common complaint in the postoperative period (5.39%). Of these, three cases (14.28%) were due to urinary tract infection, one case (4.76%) had scrub typhus, one case (4.76%) had *salmonella typhi* infection, one case (4.76%) had worm infestation as the patient had passed worms in her vomitus, and five cases (23.8%) of fever were associated with SSIs. The remaining cases may have been associated with reactionary postoperative fever. One major and fatal complication was seen on postoperative day four of abdominal myomectomy in a young female who was 25 years of age, which was a pulmonary embolism. The patient complained of sudden breathlessness with falling oxygen saturation. Four patients (1.02%) were found to have persistent tachycardia in the postoperative period up to three to four days after surgery. Two patients (0.51%) who underwent laparotomy had subacute intestinal obstruction after postoperative day four, the cause of which was unknown. They were managed conservatively after consulting with the general surgeons. Other complications observed in the postoperative period were chest infection (1.28%), abdominal distension (0.77%), urinary tract infection (0.51%), and urinary retention (0.51%).

**Table 3:** Intraoperative and postoperative complications (n=389) ECG: electrocardiogram; UTI: urinary tract infection

Complications	Number	Percentage
Intraoperative complications	23	5.91%
Intraoperative haemorrhage	14	3.59%
Urethral injury	1	0.25%
Bowel injury	1	0.25%
Bladder injury	3	0.77%
Inferior epigastric artery injury	1	0.25%
Anaphylactic shock	1	0.25%
ECG arrhythmias	2	0.51%
Postoperative complications	75	19.28%
Respiratory infection	4	1.02%
Fever	21	5.39%
Surgical site infections	40	10.28%
Urinary retention	2	0.51%
UTI	2	0.51%
Abdominal distension	3	0.77%
Subacute intestinal obstruction	2	0.51%
Pulmonary embolism	1	0.25%
Resuturing	18	4.62%

As shown in Table 4, there was a significant association between the presence of complications and parameters like age, parity, the approach of surgery, duration of surgery, preoperative duration of hospital stay (days), as well as preoperative blood transfusion.

**Table 4:** Association between complications and various parameters ***Statistically significant at p<0.05, 1: Wilcoxon-Mann-Whitney U test, 2: chi-squared test, 3: Fisher's exact test LSCS: lower segment caesarean section; BMI: body mass index; SD: standard deviation

Parameters	Complications		P-value
	Present (n=94)	Absent (n=295)	
Age (years)***, mean ± SD	43.06 ± 11.97	40.14 ± 12.96	0.0321
Parity***, n (%)			<0.0012
P0	12 (12.76%)	72 (24.4%)	
P1	3 (3.19%)	32 (10.84%)	
P2	21 (22.34%)	75 (25.42%)	
≥P3	58 (61.70%)	116 (39.32%)	
Menopausal status, n (%)			0.9322
Premenopausal	74 (78.72%)	231 (78.30%)	
Postmenopausal	20 (21.27%)	64 (21.69%)	
Comorbidities, n (%)			0.0573
Absent	74 (78.72%)	259 (87.79%)	
Hypertension	6 (6.38%)	14 (4.74%)	
Hypothyroidism	7 (7.44%)	8 (2.71%)	
Diabetes mellitus	2 (2.12%)	9 (3.05%)	
Multiple comorbidites	5 (5.31%)	5 (1.69%)	
Previous abdominal surgeries, n (%)			0.0673
None	82 (87.23%)	264 (89.49%)	
LSCS	3 (3.19%)	21 (7.11%)	
Hysterectomy	5 (5.31%)	4 (1.35%)	
Laparotomy	2 (2.12%)	3 (1.01%)	
Multiple	2 (2.12%)	3 (1.01%)	
Approach***, n (%)			<0.0012
Abdominal	78 (82.97%)	147 (49.83%)	
Laparoscopic	6 (6.38%)	89 (30.16%)	
Vaginal	10 (10.63%)	59 (20%)	
BMI (Kg/m^2^), mean ± SD	25.03 ± 5.21	25.81 ± 4.75	0.1911
BMI, n (%)			0.2993
<18.5 Kg/m^2^	7 (7.44%)	11 (3.72%)	
18.5-22.9 Kg/m^2^	30 (31.91%)	70 (23.72%)	
23.0-24.9 Kg/m^2^	14 (14.89%)	65 (22.03%)	
25.0-29.9 Kg/m^2^	28 (29.78%)	100 (33.81%)	
30.0-34.9 Kg/m^2^	11 (11.70%)	39 (13.22%)	
35.0-39.9 Kg/m^2^	4 (4.25%)	9 (3.05%)	
≥55.0 Kg/m^2^	0 (0.0%)	1 (0.33%)	
Duration of surgery***, n (%)			<0.0012
<1 hour	1 (1.06%)	34 (11.52%)	
1-2 hours	21 (22.34%)	108 (36.61%)	
2-3 hours	45 (47.87%)	107 (36.27%)	
≥3 hours	27 (28.72%)	46 (15.59%)	
Preoperative duration of hospital stay (days)***, ± SD	4.82 ± 4.21	3.80 ± 3.77	0.0041
Preoperative blood transfusion***, n (%)			0.0232
Given	17 (18.08%)	28 (9.4%)	
Not given	77 (81.9%)	267 (90.5%)	

## Discussion

“You are a true surgeon from the moment you are able to deal with your complications” - Professor Owen H Wangensteen [8].

Every surgeon tries to perform surgeries in the best possible way without any complications or harm to their patient; however, despite their best possible efforts, complications can arise in any surgery, thereby affecting the prognosis. Analysing the mistakes and maintaining a record keeps us updated about the unexpected things that can happen and helps us prepare a set of guidelines for future reference.

In our study, 389 patients were operated on by open abdominal, vaginal, and laparoscopic routes during the study period. Overall, the intraoperative or postoperative complication rate was 24.16% (94 out of the total 389 surgeries). We included all major gynaecological surgeries (including laparotomies, laparoscopic, vaginal procedures, and procedures for gynaecological cancer) so that the overall complication rate could be determined. We evaluated even minor complications such as minimal wound discharges or mild fevers requiring additional hospitalisation. Even though major complications following gynaecological surgeries are rare, if overall complications including minor problems are taken into account, they do amount to a significant rate. In light of that, we planned to analyse surgical and postoperative complications in a detailed manner through this study. The average duration of hospital stay after surgery was 10.79 + 7.91 days, and the mean duration of hospital stay was the longest in abdominal surgeries (11.5 days), followed by vaginal (7.5 days), and laparoscopic surgeries (7.3 days) due to complications. Compared to the study by Ortiz-Martínez et al. [4], where the reported prevalence of general complications following gynaecological surgery was 3.8%, our study had a higher complication rate. This could be explained by the fact that we included only major gynaecological procedures, unlike Ortiz-Martínez et al. who also analysed cases involving tubal ligation and curettage. These were less complex surgeries and hence patients were less likely to experience any complication, thereby decreasing the overall complication rate. 

Regarding the types of complications, SSIs were the most common cause of postoperative morbidity, accounting for 10.28% of cases. The prevalence of SSI varies among different studies. Ortiz-Martínez et al. [4] and Barbosa et al. [9] have reported a prevalence of 1.52% and 2.2% respectively, which are lower compared to our rates. Pathak et al. [10] in their analysis of 1,173 cases involving both obstetric and gynaecological cases reported a 10.3% SSI rate following gynaecological surgeries, which aligns with our findings. Similarly, another Indian study reported an SSI rate of 10.35% [7]. It is estimated that urogenital injuries occur in almost 1-2% of cases of major gynaecological surgeries. In our study, the frequency of urethral/bowel injury and bladder injury was 0.25% and 0.77% respectively, which is in accordance with that reported in the literature. Ortiz-Martínez et al. [4] examined the prevalence of urological injuries in gynaecological surgeries and found that bladder injury was seen in 0.34% of cases.

In our study, a significant association was seen between the presence of complications and parameters like age, parity, the approach of surgery, duration of surgery, preoperative duration of hospital stays, as well as preoperative blood transfusion. No significant association was observed with respect to previous abdominal surgeries, menopausal status, comorbidities, and BMI. Gevariya et al. [11] in their study have reported that age and parity had no relation with surgical complications. In their study, laparoscopy-assisted vaginal hysterectomy had a higher complication rate followed by abdominal hysterectomy. Erekson et al. [12] conducted a study involving a large cohort of 22,214 women, which reported the prevalence of composite 30-day postoperative complications to be 3.7%. They concluded that age of 80 years or older, medical comorbidities, dependent functional status, and unintentional weight loss were factors linked with major complications. In line with our findings, Barbosa et al. [9] and Brummer et al. [13] did not find any relationship between surgical complications and history of previous abdominal surgeries. In this study, surgical time was associated with complications, which is comparable to the findings of Ortiz-Martínez et al. [4]. In our study, a greater proportion of cases in the complication group had received preoperative blood transfusions as compared to those without complications, and the difference was statistically significant. The preoperative hospital stay duration was also longer in patients with complications compared to those without (4.82 ± 4.21 vs. 3.80 ± 3.77 days respectively, p=0.004). Our hospital caters to the needs of patients from remote hilly areas with difficult terrains and poor transportation facilities. Hence, we often have to deal with preoperative evaluation and surgery during the same admission. This prevents multiple hospital visits and minimises expenses on transportation, but increases the risk of longer preoperative hospital stays.

A major limitation of this study is that we included data from only one institution, which cannot be generalised to the general population. The major strength of our study was the inclusion of a wide spectrum of gynaecological cases that had undergone surgery through various routes. The chance of underreporting of cases is very minimal in our study as records were extracted from both operation theatre registers and from departmental Excel records. We believe that this study constitutes a unique addition to the currently available literature on surgical complications since it has included and analysed even minor complications.

## Conclusions

We believe that the best form of prevention is to avoid surgical complications altogether. Based on our findings, SSI was the most common cause of postoperative morbidity, highlighting the importance of hygiene factors. Independent parameters like age, parity, the approach of surgery, operative time, preoperative duration of hospital stay, and preoperative blood transfusion were significantly associated with perioperative complications. Further research needs to be conducted on this topic to design necessary strategies to decrease the burden on patients facing these complications.
